# A Ku-Band GaN-on-Si MMIC Power Amplifier with an Asymmetrical Output Combiner

**DOI:** 10.3390/s23146377

**Published:** 2023-07-13

**Authors:** Javier del Pino, Sunil Lalchand Khemchandani, Daniel Mayor-Duarte, Mario San-Miguel-Montesdeoca, Sergio Mateos-Angulo, Francisco de Arriba, María García

**Affiliations:** 1 Institute for Applied Microelectronics (IUMA), Universidad de Las Palmas de Gran Canaria, 35017 Las Palmas de Gran Canaria, Spain; sunil@iuma.ulpgc.es; 2 Wireless Innovative MMIC (WIMMIC), 35017 Las Palmas de Gran Canaria, Spain; daniel.mayor@wimmic.com (D.M.-D.); mario.sanmiguel@wimmic.com (M.S.-M.-M.); sergio.mateos@wimmic.com (S.M.-A.); 3 TTI, Celestia Technologies Group, 39001 Santander, Spain; farriba@ttinorte.es (F.d.A.); mrgarcia@ttinorte.es (M.G.)

**Keywords:** gallium nitride (GaN), GaN on silicon (GaN-on-Si), high-power amplifier (HPA), Ku band

## Abstract

In this paper, a microwave monolithic integrated circuit (MMIC) high-power amplifier (HPA) for Ku-band active radar applications based on gallium nitride on silicon (GaN-on-Si) is presented. The design is based on a three-stage architecture and was implemented using the D01GH technology provided by OMMIC foundry. Details on the architecture definition and design process to maximize delivered power are provided along with stability and thermal analyses. To optimize the amplifier performance, an asymmetry was included at the output combiner. Experimental results show that the HPA achieves a 39.5 dBm pulsed-mode output power, a peak linear gain of 23 dB, a drain efficiency of 27%, and good input/output matching in the 16–19 GHz frequency range. The chip area is 5 × 3.5 mm${}^{2}$ and for the measurements was mounted on a custom-made module. These results demonstrate that GaN-on-Si-based Solid-State Power Amplifiers (SSPAs) can be used for the implementation of Ku-band active radars.

## 1. Introduction

Active radars worldwide operate in various frequency bands, including the X, C, L, S, and Ku bands, which can be combined for certain applications. The L and S bands are commonly used in dense environments such as forests or glaciers due to their penetration capabilities. The X and C bands, on the other hand, are employed for high-resolution applications, making them valuable for tactical purposes. Additionally, the Ku band is particularly advantageous for studying large surfaces and achieving higher resolutions, making it popular in military applications [1].

The Ku band offers benefits such as enabling the use of small antennas in lightweight applications like Synthetic Aperture Radar (SAR), Surface Movement Radar (SMR), Ground Surveillance Radar (GSR), and Altimeter Radar. These radar systems often require peak power ranging from a few watts to tens of watts, utilizing short pulses to achieve high accuracy, high update rates, and high-resolution target detection [2,3,4,5,6,7].

To meet the demands of these applications, the development of high-power amplifiers (HPAs) is essential. HPAs must generate high RF power at the operating frequency while maintaining enhanced efficiency. While efficiency and output power are important, ensuring high reliability and controlled aging for long-term operation is equally crucial. This necessitates optimizing circuit designs to operate at reduced levels of electrical and thermal stress. The primary source of stress for high-power active devices is self-heating, which calls for lowering the maximum operating temperature of these devices compared to the technology’s maximum rating.

Achieving high output power in an HPA often requires a large number of transistors in parallel in the final stage [8,9,10,11,12,13]. To enhance reliability and extend the HPA’s lifespan, it is necessary to ensure that the transistors operate below the maximum power density allowed by the technology. Consequently, increasing the number of transistors in parallel in the final stage becomes necessary to ensure they operate well below the technology’s maximum power density. However, designing the output combiner becomes more challenging as the number of transistors increases.

In this paper, we propose the use of an asymmetrical output combiner for designing a 16–19 GHz GaN high-power amplifier (HPA) specifically for active radar applications. Thanks to this asymmetry, all the output transistors are matched closer to their optimum load impedance, resulting in all of them providing nearly the same performance.

The organization of this paper is as follows: Section 2 discusses the general characteristics of the employed technology, the OMMIC’s D01GH GaN-on-Si process. Section 3 focuses on the design of the HPA, while Section 4 presents the measurement results. Finally, Section 5 provides the conclusions derived from this study.

## 2. Technology Description

The D01GH process is based on an AlN-GaN-AlGaN Double Heterostructure Field-Effect Transistor (DHFET) active layer, which can be fabricated on either a high-resistivity silicon substrate (GaN-on-Si) or a silicon carbide substrate (GaN-on-SiC). GaN-on-SiC devices generally offer higher output power ($P_{out}$), power density, and power-added efficiency (PAE) than GaN-on-Si devices. Furthermore, the SiC substrate exhibits lower losses and better thermal conductivity compared to the Si substrate. However, the SiC substrate is more expensive to manufacture and is not compatible with other Si-based technologies. In this design, the silicon substrate was chosen due to its availability.

The transistors of this technology feature a mushroom-shaped 100 nm gate to enhance performance in terms of noise and frequency. The kit includes non-alloyed ohmic contacts for ultra-low resistance and transistor-based diodes for mixing, level shifting, or implementing varactors. Two different ways to implement resistors and Metal–Insulator–Metal (MIM) capacitors can be utilized, depending on which layers are involved.

The process is intended to be used for microwave and millimeter-wave applications and includes air bridges, thick interconnect metal, and high resistivity via holes. This process utilises in situ passivation to minimize lag effects [13].

The main electrical characteristics of this process include a cut-off frequency of 105 GHz and an RF power density of 3.3 W/mm (with a peak value of 5.7 W/mm) [13]. The breakdown voltage is 40 V, and the quiescent V_DD_ is 12 V.

## 3. HPA Design

**Figure 1:**
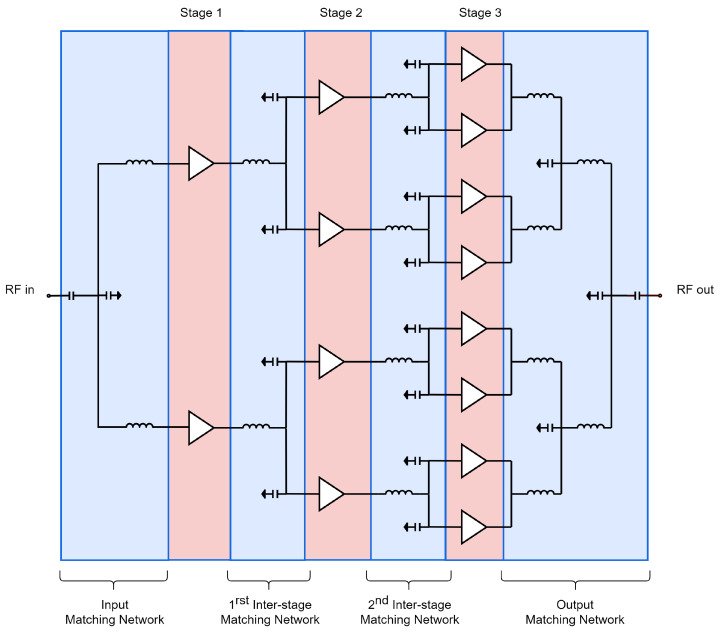
HPA architecture.

**Figure 2:**
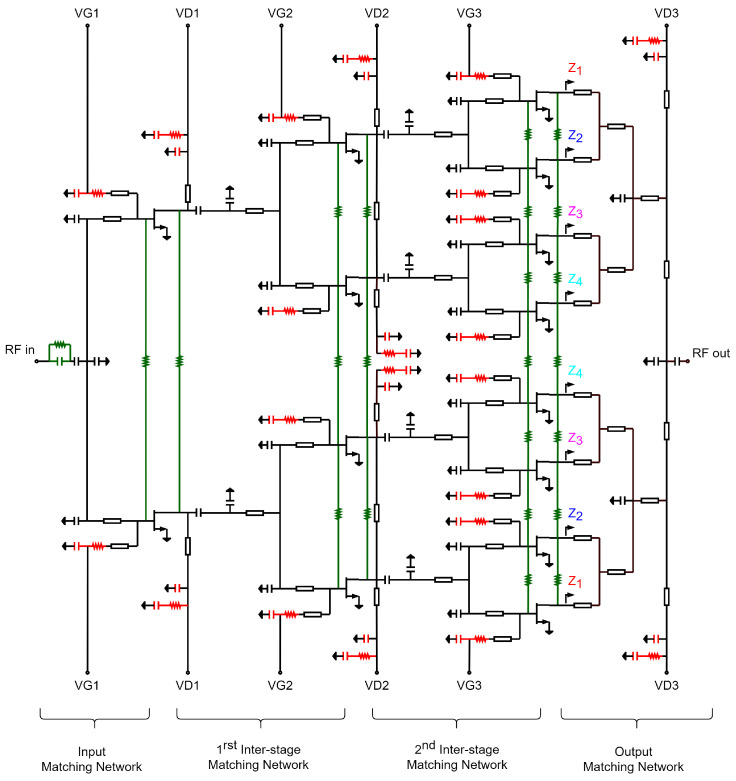
HPA simplified schematic.

**Figure 3:**
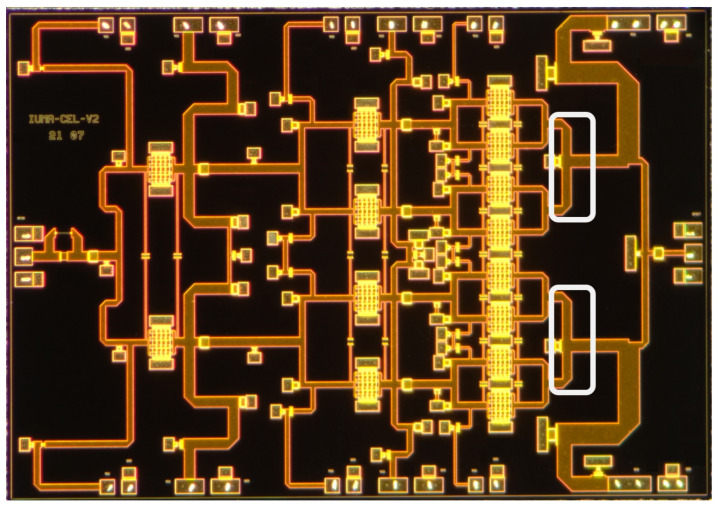
Microphotograph of the circuit with the asymmetry at the output stage in gray.

The first step in designing an HPA is to define its architecture, including the number of stages and the number of transistors in each stage. The Load/Source pull method [14] based on the process design kit (PDK) devices was used to optimize the size of the transistors for the desired performance. A specific transistor with eight fingers and a finger width of 125 µm was chosen for its good saturated power and linear gain: approximately 32 dBm and 10 dB, respectively. To achieve a gain of approximately 25 dB, the number of stages was set to three, taking into account the insertion losses introduced by the interstages that connect the different amplification stages. In order to ensure a long lifespan for the circuit while achieving a theoretical output power of 41 dBm, we employed eight transistors in the last stage with a power density of 1.58 W/mm. This power density is significantly lower than the maximum allowed by the technology (3.3 W/mm). By operating under these conditions, the transistors remain well below the maximum temperature of 200 °C specified by the technology for a backside temperature of 85 °C. Finally, to ensure proper signal distribution to the last stage, the second and first stages were composed of four and two transistors, respectively. Figure 1 shows the overall architecture of the HPA, while Figure 2 and Figure 3 display the schematic and a die photograph of the manufactured circuit, respectively. The design occupies an area of 5 × 3.5 mm${}^{2}$. In the following subsections, a brief description of the HPA design is presented.

### 3.1. Matching Networks Design

As shown in Figure 2, the output matching network is an interface between the eight transistors of the third stage (power stage) and the ground–signal–ground (GSG) output port. Its main purpose is to provide the optimum load to the transistors while minimizing the losses. The design of this network is the most critical part when designing an HPA, since the output power level directly depends on it. The output matching network was designed to be symmetrical to the output of the circuit and originally consisted of a double-L matching network made up of two T-lines and two capacitors. Thanks to the symmetry of the network, only three capacitors were needed for the entire matching network, instead of the sixteen originally needed (two for each transistor).

**Figure 4:**
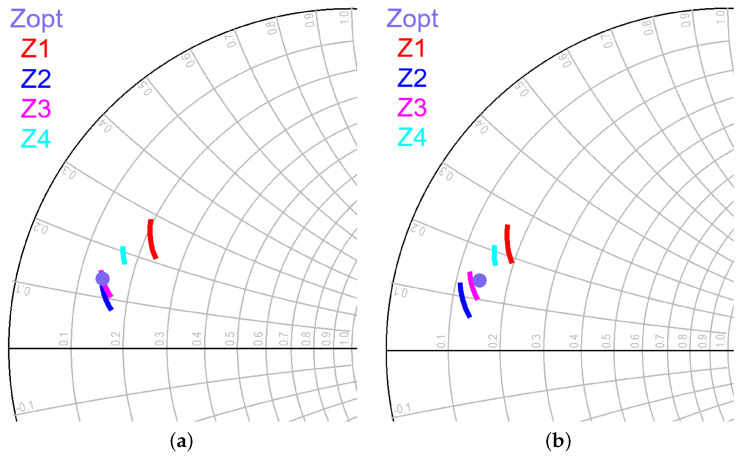
Output stage matching: (**a**) before asymmetry; (**b**) after asymmetry.

**Figure 5:**
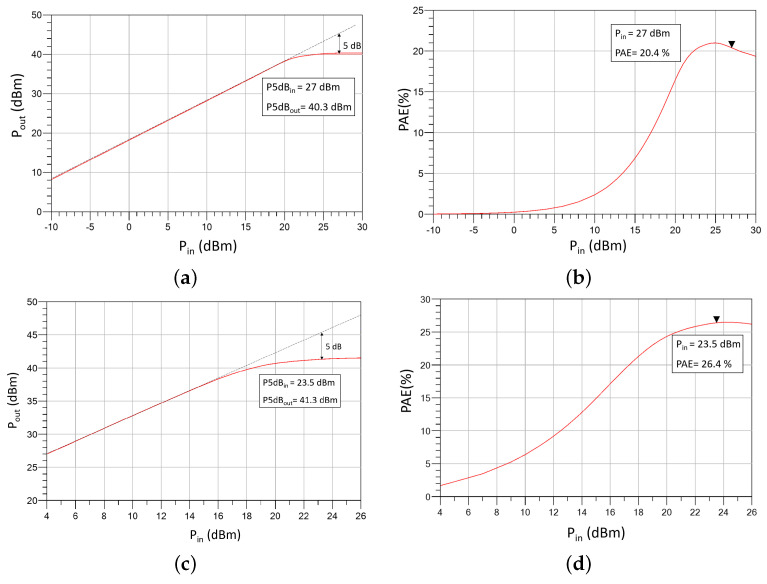
Simulated $P_{out}$ and PAE as a function of $P_{in}$: (**a**,**b**) before asymmetry; (**c**,**d**) after asymmetry.

As shown in Figure 3, a slight asymmetry was included in the layout of the output matching network by making the output T-lines slightly different in length. This was because EM simulations revealed that if the output network is completely symmetrical, the coupling between the lines results in different loadings for each of the eight output transistors. This means that not all transistors in the output stage contribute to the output power in the same way. To illustrate this, Figure 4 displays the optimum impedance for maximum power delivery (Zopt) and the actual impedances that the top four transistors see in the 17.3 GHz to 18.4 GHz frequency range after analyzing the EM simulations of the output matching network ($Z_{1-4}$) for the cases when the output network is completely symmetrical (Figure 4a) and when the output network has the asymmetry shown in Figure 3 (Figure 4b). It can be observed that with a symmetric output matching network, $Z_{2}$ and $Z_{3}$ are close to Zopt, while $Z_{4}$ and $Z_{1}$ deviate significantly (see Figure 4a). However, after including the asymmetry, impedances $Z_{1-4}$ are uniformly distributed around Zopt (see Figure 4b). This significantly improves the large-signal response, as shown in Figure 5, where the simulated $P_{sat}$ at 5 dB compression and the PAE of the design without asymmetry (Figure 5a,b) and with asymmetry (Figure 5c,d) are displayed. The simulations demonstrate that the inclusion of asymmetry results in a 1 dBm improvement in Pout and a 6% improvement in PAE.

The procedure followed to develop the rest of the matching networks was the same as explained above. The main difference is that the objective when designing output matching networks is to minimize insertion loss, while in the case of inter-stage matching networks the goal is mainly to minimize mismatch loss, and in the input matching network it is to improve input return loss. To achieve these objectives, EM simulations and optimization were used.

### 3.2. Stability Analysis

When designing an HPA, the appearance of oscillations related to circuit instabilities must be carefully avoided. Different types of oscillations can occur in microwave power amplifiers: low frequency or bias, even mode or small signal, odd mode and parametric [15].

In the drain biasing networks, series RC filters in parallel with de-coupling capacitors were included to remove low-frequency stability issues. The capacitors were sized to reduce oscillations at low frequencies, in this case around 2 GHz.

The inherent stability of a transistor itself is known as even-mode stability. Even-mode oscillations occur when the load or source impedances connected at the transistor input or output provide a reflection coefficient magnitude greater than one. This is usually avoided by adding a stabilization circuit. In this case, a parallel RC circuit at the input of the amplifier was used to remove even-mode instabilities.

Odd-mode oscillations are due to the instabilities that could appear in a transistor due to leakage caused by its connection in parallel with another transistor [14]. To prevent such oscillations, stabilization buses made up of small resistors, called isolation resistors, connected between the input and the output of the transistors were included.

Finally, parametric oscillations occur when the power amplifier enters the nonlinear regime. In this situation, the transistors’ internal nonlinear elements vary, generating unstable feedback loops. The conventional method to detect parametric oscillations is to calculate the zero-poles of all closed-loop transfer functions in the nonlinear regime. The amplifier is unstable if there is a pole with a positive real part located in the right half plane (RHP) of one transfer function [16]. Parametric oscillations are associated with an increase in gain when the power amplifier enters the nonlinear regime. The most common way to reduce these oscillations is to place a resistor in series with the gate of one transistor to reduce the gain. In our case, the input parallel RC circuit used to remove even-mode instabilities also removed parametric oscillations.

### 3.3. Thermal Analysis

GaN technology can achieve high power densities, which can lead to increased self-heating and higher operating channel temperatures in the transistor. This is especially problematic in GaN technologies that use a silicon substrate, due to its poor thermal conductivity. The thermal resistance of GaN-on-Si technology is about twice that of GaN-on-SiC [17], which means that the maximum power that can be dissipated is much lower and, therefore, the achievable power density will also be lower.

**Figure 6:**
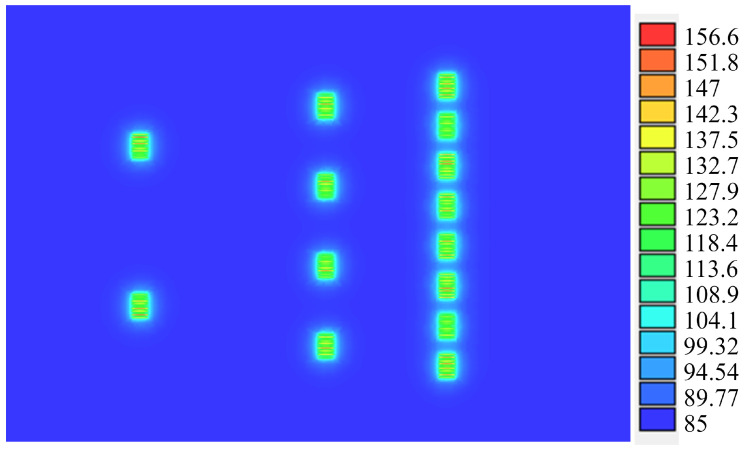
Temperature analysis using ADS electrothermal simulator.

To ensure that the circuit will stay below the maximum junction temperature (Tj) set by the foundry to prevent malfunctions (200 °C), a thermal analysis was performed using the ADS Electrothermal Simulator assuming a backside temperature of 85 °C. As can be observed in Figure 6, the maximum value of the temperature is 156.6 °C, which is reached at the gates of the transistors of the last stage.

## 4. Measurement Results

**Figure 7:**
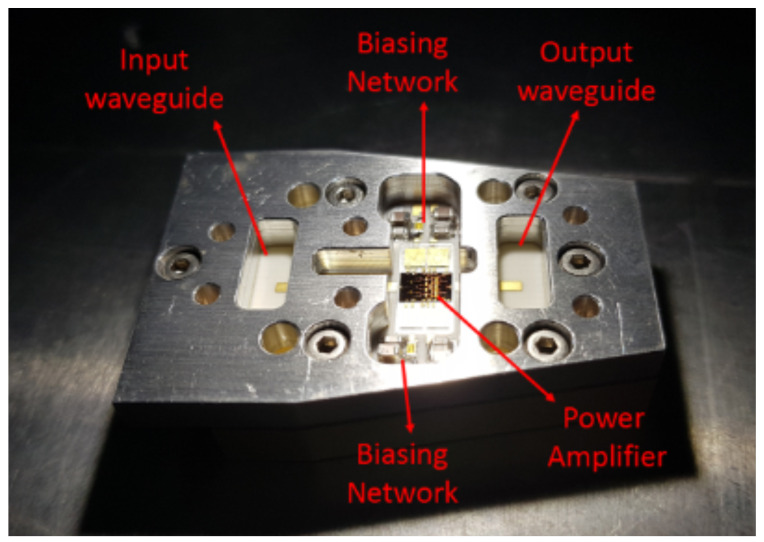
Measurement module.

Figure 7 shows the measurement module without the lid. This includes the HPA and the external bias networks. The cavity resonance coupled with the HPA can draw a huge quiescent current, which can cause the breakdown of the device. Also, the resonance can affect the gain, stability, $P_{out}$, etc. [18]. To mitigate these effects, an RF absorber with a height of 0.8 mm was included on the lid of the module. The signals were inserted and extracted using waveguides. A Peltier thermoelectric module was used to refrigerate the circuit.

**Figure 8:**
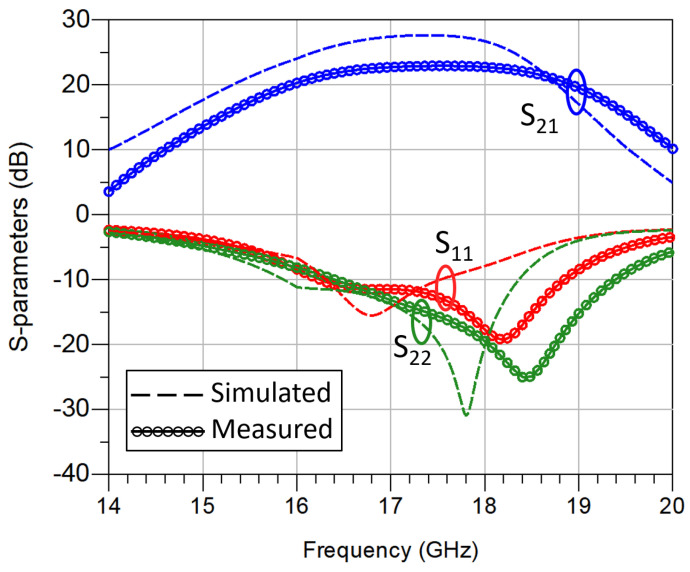
Power amplifier measurement results: S-parameters.

**Figure 9:**
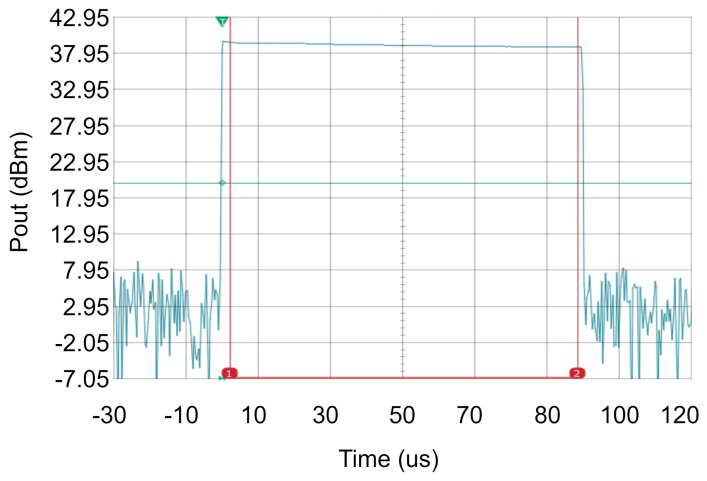
Power amplifier measurement results: pulsed output power at 17.3 GHz, pulse width = 100 µs, pulse duty cycle = 1%, $V_{DS}$ = 11 V, $I_{DC}$ = 3.3 A.

The S-parameter measurements were carried out in continuous-wave mode and the results are shown in Figure 8. For $V_{DS}$ = 10 V, the measurements show that the $S_{21}$ is above 20 dB from 16 GHz to 19 GHz, reaching a peak gain of 23 dB at 17.6 GHz. Secondly, the $S_{11}$ parameter is below –10 dB from 16.3 GHz to 18.8 GHz. Regarding the output matching ($S_{22}$), its value is below –10 dB from 16.4 GHz to 19.3 GHz. The power measurements were performed in pulsed mode using 100 µs pulses and a duty cycle of 1%. Figure 9 shows the saturated output power for the designed HPA at 17.3 GHz. This circuit provides 39.5 dBm of output power, which is equivalent to 8.9 W. These results were achieved under a total current consumption of 3.3 A and 11 V of drain voltage. The drain efficiency was measured. Since the gain of the circuit is large, the PAE can be considered similar to the drain efficiency. The resulting drain efficiency is 27%, with $P_{out}$ = 8.91 W, $V_{DC}$ = 11 V, and $I_{DC}$ = 3.3 A. An amplifier module based on this MMIC would meet both short-pulse (20–100 ns) and long-pulse (1–100 µs) applications with an output power between 8 and 100 W if various MMICs were combined.

Table 1 shows a comparison of state-of-the-art composite GaN power amplifiers using OMMIC’s 100 nm GaN-on-Si technology. The designs in [10,11] outperform ours in terms of PAE and output power, but they achieve this by driving their transistors to the maximum power density allowed by the technology. In contrast, our design prioritizes reliability and longevity by sacrificing some power to keep the transistors operating well below 200 °C (assuming a backside temperature of 85 °C). The thermal simulator indicates that our maximum temperature is only 156.6 °C, which provides a much longer lifespan for our design. In terms of area, our design occupies a larger footprint due to its lower operating frequency. Although [10] operates in a similar frequency range, it uses a smaller area because it has only two stages and consequently achieves a lower gain.

## 5. Conclusions

In this paper, a composite MMIC HPA for Ku-band active radar applications based on OMMIC’s D01GH GaN-on-Si process is presented. To ensure that all transistors in the output stage contribute to the output power in the same way, an asymmetry was added to the output combiner. The HPA achieves a peak gain of 23 dB, and good input/output matching in the 16–19 GHz frequency range, with a pulsed-mode output power of 39.5 dBm and a drain efficiency of 27% for a pulse width of 100 µs. These results support the use of GaN-on-Si-based SSPAs for the implementation of Ku-band active radars.

## Figures and Tables

**Table 1:** Comparison with other OMMIC 100 nm GaN-on-Si PAs.

Ref.	Freq. (GHz)	N. Stages	Gain (dB)	PAE (%)	P_out_ (dBm)	V_d_ (V)	P. dens. (W/mm)	Size (mm${}^{2}$)
[8]	31–35	4	27	20	35	9	1.05	3.7 × 2
[9]	35–36.5	4	20	26	36	9	1.33	–
[10]	6–18	2	20	48	41	12	3.03	4.2 × 3.1
[11]	36–44	3	20	35	41.3	12	3.24	3.6 × 2.8
[12]	45.5–47.2	4	17.5	–	37	12	1.23	3.7 × 2.8
[13]	27–34	3	29	32	37.5	12	1.76	4.5 × 3.5
This Work	16–19	3	23	27	39.5	11	1.11	3.5 × 5

## Data Availability

Not applicable.

## Abbreviations

The following abbreviations are used in this manuscript:

DHFET	Double Heterostructure Field-Effect Transistor
EM	Electro Magnetic
GaN	Gallium Nitride
GaN-on-Si	Gallium Nitride on Silicon
GaN-on-SiC	Gallium Nitride on Silicon Carbide
GSG	Ground–Signal–Ground
GSR	Ground Surveillance Radar
HPA	High-Power Amplifier
MIM	Metal–Insulator–Metal
MMIC	Microwave Monolithic Integrated Circuit
PAE	Power-Added Efficiency
PDK	Process Design Kit
RC	Resistor Capacitor
RF	Radio Frequency
RHP	Right Half Plane
SAR	Synthetic Aperture Radar
SiC	Silicon Carbide
SMR	Surface Movement Radar
SSPA	Solid-State Power Amplifier
